# Trends of Esophageal Cancer Mortality in Rural China from 1989 to 2013: An Age-Period-Cohort Analysis

**DOI:** 10.3390/ijerph14030218

**Published:** 2017-02-23

**Authors:** Xudong Gao, Zhenkun Wang, Chan Kong, Fen Yang, Ying Wang, Xiaodong Tan

**Affiliations:** 1 Department of Occupational and Environmental Health, School of Health Sciences, Wuhan University, 115 Donghu Road, Wuhan 430071, China; gaoyirune@yeah.net (X.G.); wongzhenkun@gmail.com (Z.W.); lazydove@126.com (Y.W.); 2 Department of Nursing, College of Health Science & Nursing, Wuhan Polytechnic University, 68 Xuefunan Road, Wuhan 430023, China; 3 Comprehensive Medical Department, Tongji Hospital of Tongji Medical College of HUST, 1095 Jiefang Avenue, Wuhan 430030, China; kongchan_625@126.com; 4 Department of Nursing, School of Nursing, Hubei University of Chinese Medicine, 1 Huangjiahuxi Road, Wuhan 430065, China; alextobbj@163.com

**Keywords:** esophageal cancer, mortality, APC, rural China

## Abstract

*Background*: Esophageal cancer is one of the most common cancers in rural China. The aim of this study was to describe the time trends of esophageal cancer mortality in rural China and to better elucidate the causes of these trends. *Methods*: The mortality data were obtained from the World Health Organization Mortality Database and the China Health Statistical Yearbook Database. The mortality data were analyzed with age-period-cohort (APC) analysis. *Results*: Our study indicates that the Age-Standardized Mortality Rates (ASMRs) in rural China generally decreased from 1989 to 2003, and thereafter increased until the year 2008 in both sexes. After 2008, the ASMRs decreased again. The results of APC analysis suggest that the general decrease in esophageal cancer mortality in rural China from 1989 to 2003 might be caused by the downtrend of the cohort effects and period effects, while the general increase in mortality from 2004 to 2008 might be caused by the uptrend of the period effects. The decrease in mortality after 2008 may be relevant to the Four Trillion RMB Investment Plan launched by the Chinese Government. *Conclusions*: The declining cohort effects were probably related to the improvement of socioeconomic status in childhood and the decreasing consumptions of alcohol drinking and smoking, while the trends of the period effects were relevant to the changes in the dietary pattern. Our findings may help predict future changes in esophageal cancer mortality.

## 1. Introduction

Esophageal cancer is relatively infrequent in Europe and America, but worldwide it is the sixth deadliest cancer [1]. Esophageal cancer affects more than 450,000 people worldwide, and mortality associated with esophageal cancer is increasing rapidly [2]. Esophageal squamous cell carcinoma (ESCC) and esophageal adenocarcinoma (EAC) have been defined as two main histologic types of esophageal cancer. EAC has emerged as the major histologic type in some Western countries [3], while ESCC is the predominant histologic type in the so-called Asian belt, which includes Turkey, Iran, and China [2].

China accounts for nearly 20% of the world population, but almost half of the total esophageal cancer deaths in the world [1]. In China, esophageal cancer is the fourth most common cancer, and the associated mortality varies largely between rural and urban areas [4]. In general, esophageal cancer mortality in rural China is significantly higher than in urban China [4]. More worrying, according to the China Health Statistical Yearbook Database, the esophageal cancer mortality in rural China showed an upward tendency in recent years [5]. The Age-Standardized Mortality Rate (ASMR), per 100,000 male population for esophageal cancer in rural China increased from 14.4 in 2002 to 24.5 in 2013 [5]. Unfortunately, the reason for this trend is still unknown, because of the lack of relevant studies.

Age-period-cohort (APC) analysis aims to describe the secular trends in disease by separately assessing the effects of age, period, and birth cohort on mortality. It is a popular analytical method in both epidemiological and sociological studies [6,7], and it has been applied to analyze the character and quality of trends in the prevalence of many diseases, such as liver disease, cardiovascular disease, and many kinds of cancer.

Several studies have adopted APC analysis to assess trends of esophageal cancer in some areas of China (e.g., Shanghai [8], Hong Kong [9], Linxian [10], Dalian [11]). However, to our knowledge, there are no published studies that focus exclusively on the trend of esophageal cancer mortality in rural China using APC analysis. In order to describe the secular trends of esophageal cancer mortality in rural China, and to better explain the underlying causes of these trends, we applied the APC model to analyze the separate effects of age, period, and birth cohort on esophageal cancer mortality. 

First, the mortality trends of esophageal cancer in rural China were described. Second, the APC model was used to analyze differences in the effects of age, calendar period, and birth cohort on mortality trends. Last, we explored possible reasons for the observed mortality trends. It is interesting and important to examine how the mortality trends in rural China were influenced. Understanding the underlying reasons for changing trends in mortality associated with esophageal cancer will generate useful hints for the prevention of esophageal cancer in rural China.

## 2. Materials and Methods

### 2.1. Source of Data

The esophageal cancer mortality data from the years 1989 to 2000 were available from the World Health Organization Mortality Database. The mortality data from the years 2001 to 2013 were available from the China Health Statistical Yearbook Database [5,12,13]. It should be noted that both databases were created by the Centre of Health Information and Statistics of China, thus they were considered to be combined to achieve a longer time span [5]. The esophageal cancer mortality data in both databases were divided into rural areas and urban areas. The data included in our study were obtained from the rural areas. The China Health Statistical Yearbook Database defines the word “rural” as the geographic area that is located outside of cities, including counties, townships, and villages in China [5]. Esophageal cancer cases aged 30–84 years old were included in this study, because of the fact that patients with esophageal cancer under the age of 30 is very rare, and patients above the age of 85 usually died from competing causes, such as acute myocardial infarction, acute pulmonary embolism, and cute respiratory distress syndrome [8,14].

### 2.2. Statistical Analysis

In order to describe the trends of esophageal cancer mortality in rural China, the mortality rates from 1989 to 2013 were age-standardized, using the world standard population proposed by Segi and modified by Doll [15,16]. These mortality rates were smoothed with 5-year moving averages to reveal secular trends and decrease short-term fluctuations. For the APC analysis, the esophageal cancer data in this study were collected in five successive five-year periods from 1989–1993 to 2009–2013, and 11 five-year age groups, ranging from 30–34 years to 80–84 years. 

The APC model represents a classic epidemiological approach that can be used to extract information from cross-sectional data regarding historical changes in mortality and morbidity risk. The APC model is categorized into multiple regression models and can be expressed as:

*Y* = *µ* + *αX*_1_ + *βX*_2_ + *γX*_3_ + *ε* (1)

where *X*_1_, *X*_2_, *X*_3_ represent age, period, cohort, respectively, and *α*, *β*, *γ* represent their parameter estimates, respectively.

When it refers to the estimation of cancer mortality data, the model can be written as a log-linear Poisson model:

*ln*[*E*(*M_ij_*)] = *ln* (*D_ij_*/*P_ij_*) = *µ* + *α_i_* + *β_j_* + *γ_k_* + *ε_ij_* (2)

where *E*(*M_ij_*) denotes the expectation of death rate for the *i*th age group for *i* = 1, . . . , *a* age group at the *j*th time period for *j* = 1, . . . , *p* time period born in the *k*th cohort for *k* = 1, . . . , *α* + *p* − 1 birth cohort of observed data; *M_ij_*, *D_ij_* and *P_ij_* denote the observed death rate, death number and size of population of that group, respectively; *µ* denotes the intercept or adjusted mean death rate; *α_i_* denotes the *i*th row age effect or the coefficient for the *i*th age group; *β_j_* denotes the *j*th column period effect or the coefficient for the *j*th time period; *γk* denotes the *k*th diagonal cohort effect or the coefficient for the *k*th cohort; *ε_ij_* denotes the random error with expectation *E*(*ε_ij_*) = 0.

However, one longstanding problem associated with the APC analysis, there is a linear relationship between the age, period, and cohort (Period = Age + Cohort) [6]. It is difficult to analyze the unique set for every age, period and cohort effect, which is called as the non-identification problem [6,17]. To address this problem, we utilized the Intrinsic Estimator (IE) algorithm put forward by Fu [18] which is a novel and promising method. The IE algorithm applies the estimable functions and the singular value decomposition of matrices to approach the estimator of the APC model [18]. We chose the IE method because of its superior estimation ability, non-biased approach, validity, and asymptotic features [16,18,19]. The goodness-of-fit of the model was assessed by comparing the fit to the Akaike information criterion (AIC) and the Bayesian information criterion (BIC). For data processing, we used Microsoft Excel (Microsoft, Redmond, WA, USA) and Stata version 12.0 (StataCorp, College Station, TX, USA).

## 3. Results

**Figure 1:**
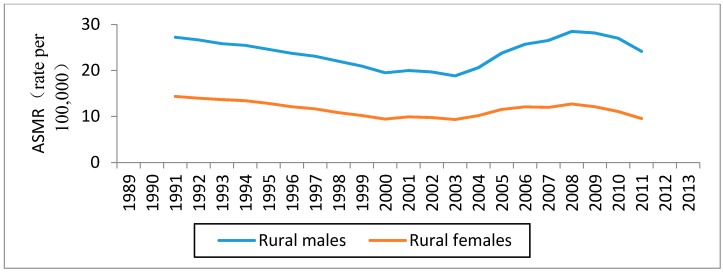
Trends in the five-year moving average age standardized mortality rates per 100,000 population for esophageal cancer in rural China.

Age-specific mortality rates for esophageal cancer by year of death in rural China were listed in Table A1 (see Appendix A). Figure 1 shows the trends of the ASMRs for esophageal cancer in rural China, by sex for the period of 1989–2013, using five-year moving averages. Higher mortality rates were observed among men than among women, in accordance with studies in other parts of the world [20]. The ASMR of the female population was no more than 15/100,000 people throughout the observation period. The ASMRs in rural China generally decreased from 1989 to 2003, and thereafter increased until the year 2008 in both sexes. After 2008, the ASMRs in rural China decreased again.

**Figure 2:**
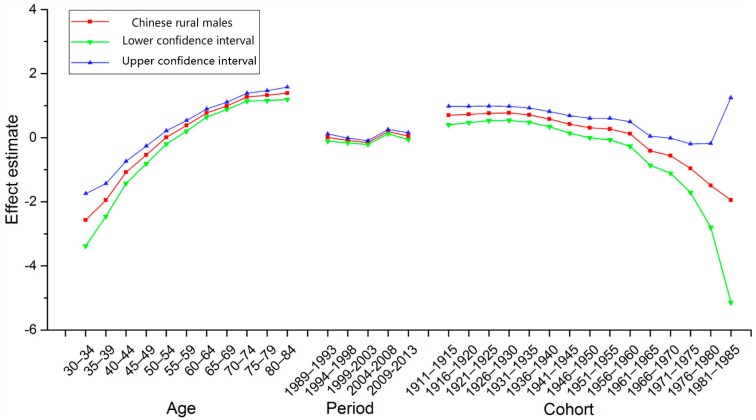
Age, period, and cohort effect on esophageal cancer mortality among men in rural China and the corresponding 95% confidence intervals.

**Figure 3:**
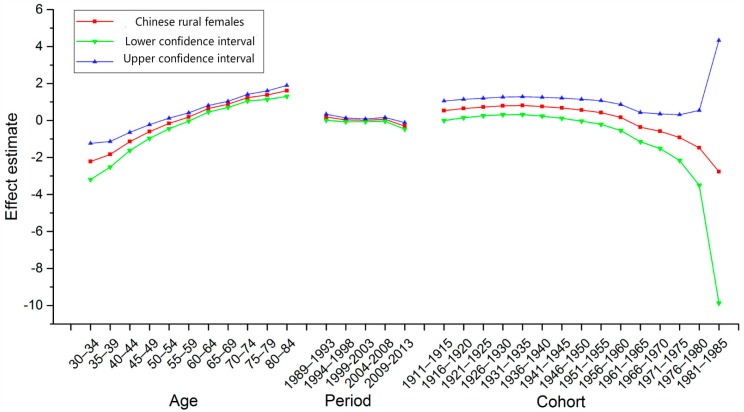
Age, period, and cohort effect on esophageal cancer mortality among women in rural China and the corresponding 95% confidence intervals.

Goodness-of-fit was evaluated synthetically with AIC and BIC, as shown in Table A2 (See Appendix A). These models, which utilize intrinsic estimator algorithm, were the best fitted to describe the trends in esophageal cancer mortality in our study. The specific results of APC model analysis were listed in Table A3 (See Appendix A). Figure 2 and Figure 3 show the trends of the age, period, and cohort effects in rural China during the observation period, by sex. The Y-axis for each effect represents the natural logarithm of the relative mortality rate of esophageal cancer at the corresponding age, calendar year of death, or birth year of the patients. The features of these trends in each effect were described as below.

*Age Effect*. The age effect in rural China increased approximately linearly with increasing age from 30 to 59 years of age in both sexes. After 59 years of age, the age effect continued to rise, peaking at approximately 84 years.

*Period Effect*. The period effect in rural China decreased from 1989 to 2003, and thereafter generally increased until the year 2008 in both sexes. After 2008, the period effect decreased again. 

*Cohort Effect*. The cohort effect in rural China generally decreased from the 1910s to the 1980s. In both sexes, the cohort effect maintained a relatively stable trend before 1935, which was followed by a decline. The cohort effect declined at an accelerated rate since the 1950s. The downtrends showed a sharp decrease in the youngest generation, though it should be noted that there was less data available for the younger age groups compared with other age groups.

## 4. Discussion

The patients’ age, birth year, and calendar year of cancer death are three factors that can affect the mortality trend of cancer. In general, period effects are typically interpreted as environmental changes that affect all age groups, as well as advancements in treatment and diagnosis [13]. Cohort effects reflect prolonged exposure in early life, such as life-style or environmental toxins, which will manifest them as a progressive increase or decline in cancer risk when changes emerge within the population [13]. To be clear, not all risk factors for esophageal cancer were included in APC analysis. Only risk factors related with the trends of age, period, and birth cohort effects were discussed. In both sexes, the ASMRs in rural China showed a general decrease from 1989 to 2003, and thereafter the ASMRs showed an increase until 2008. Based to the results of the APC analysis, we presumed that the general decrease in esophageal cancer mortality in rural China from 1989 to 2003 was caused by the downtrend of the cohort effects and period effects, while the general increase in mortality from 2004 to 2008 was caused by the uptrend of the period effects. 

The ASMRs of esophageal cancer revealed trends in esophageal cancer mortality in rural China during the observation period. Our findings were consistent with the results of Lin et al. [4], which revealed mortality trends in esophageal cancer in rural China and urban China. Higher esophageal cancer mortality was observed among men than among women consistent with other studies. It has proved that gender differences in esophageal cancer mortality are thought to be related to differences in sex hormones and lifestyle [1,9,11,20]. The age effect on mortality rates of esophageal cancer in rural China showed progressively increasing trends. This indicates that elderly people have a higher risk of esophageal cancer, which may be because of long-term exposure to carcinogenic substance. This result comes as no surprise to most of the clinicians and scholars. Of more interest is the fact that the results of our APC analysis indicate that the cohort effect and the period effect also played an important role in esophageal mortality trends. 

The cohort effect for the very young and very old generations should be explained carefully, because of the relatively small number of observations upon which they are based; they have greater standard errors than estimates for the middle cohorts [18]. For this reason, the general trends of the cohort effect in the middle range were focused in our study. The cohort effects in the middle range showed a general downward trend. Additionally, it is worth noting that the cohort effects in both sexes showed a consistently accelerated decline from the 1950s generation. 

The downtrend in the cohort effects were similar to other studies that applied APC analysis to esophageal cancer mortality in Shanghai [8] and Linzhou [10] as well as the esophageal cancer incidence in Hong Kong [9] and Dalian [11], whereas using different analytical methods. The general decrease in ASMRs of esophageal cancer in rural China from 1989 to 2003 might be caused by the downtrend of the cohort effects. There is no definite explanation for the cause of the decreasing cohort effects. However, the improvement of socioeconomic status (SES) during childhood in these areas may partly explain the result. It has been proved that individuals with adverse SES in childhood had a shorter length of life and higher mortality, independent of socioeconomic conditions later in life [21,22]. Socioeconomic conditions and public health conditions in rural China were poor in the early 1900s, but the economic development in rural China after World War II improved the situation [23]. The economic growth in rural China may explain the accelerated decrease of the cohort effects from those born since the 1950s. Two hypotheses for the potential mechanisms are likely to support this correlation. First, the improvement of education level with socioeconomic development resulted in increased awareness of esophageal cancer. Second, adverse SES in childhood may lead to higher biological susceptibility to esophageal cancer. 

One of the most reasonable explanations for the observed decline in cohort effects is the corresponding decrease in prevalence of tobacco and alcohol consumption in younger generations. A study conducted in the United States found that childhood SES was adversely related to tobacco and alcohol use in their later life [24]. Tobacco smoking and alcohol drinking have long been recognized as risk factors for both ESCC and EAC [9]. Additionally, the consumption of both tobacco and alcohol results in a synergistic effect in increasing the risk of developing esophageal cancer [25]. 

One study on the prevalence of smoking tobacco among the Chinese rural population of different generations demonstrated that the prevalence of smoking decreased gradually from those born in the 1950s through those born in the 1980s [26]. Also, the constant decrease in the rate of frequent alcohol drinking in rural China from those born in the 1940s may partly impact the decline in cohort effects [27]. Considering on a reasonable latency period between exposures and esophageal cancer mortality, the progressive protective cohort effects in more recent generations might be related to the decreasing levels of tobacco and alcohol consumption in rural China.

The absolute changes in period effects in China from 1989 to 2013 were relatively small. However, as shown in Figure 1, Figure 2 and Figure 3, the trends of the ASMRs were almost consistent with the trends of the period effects in rural China. Therefore, we speculated that period effects might also influence esophageal cancer mortality trends in rural China. The downtrend in the ASMRs of esophageal cancer in rural China from 1989 to 2003 might be caused by the decrease of the period effects from 1989 to 2003. Similarly, the uptrend in the ASMRs from 2004 to 2008 might be caused by the increase of the period effects from 2004 to 2008. Major risk factors for esophageal cancer in rural China have been suggested to be associated with particular dietary habits, such as high consumption of pickled vegetables and salty food, low intake of fresh vegetables and fruits, and drinking beverages at high temperatures [14,28].

The National Nutrition and Health Survey by the China’s Health Ministry, which reported diet status in rural China from 1982 to 1992, showed that daily intake of dark color vegetables increased from 84.0 g to 107.1 g; daily intake of fruits increased from 37.0 g to 49.0 g; daily intake of pickled vegetables decreased from 14.8 g to 10.8 g [29,30]. In view of the latency time between exposures and esophageal cancer onset, the decreasing period effects from 1989 to 2003 might be related to the improvements in the dietary pattern of the Chinese rural population from 1982 to 1992, including increased consumption of vegetables and fruits, and decreased intake of pickled vegetables. Apart from the changes in dietary pattern, other possible reasons for the decrease in the period effects in the 1990s are: improvements in esophageal cancer treatment [11,31] and increasing the use of endoscopy in diagnosis [31].

According to the National Nutrition and Health Survey, the daily intake of dark color vegetables decreased from 107.1 g in 1992 to 92.8 g in 2002; the daily intake of fruits decreased from 49.0 g in 1992 to 45.0 g in 2002; the daily intake of pickled vegetables increased from 10.8 g in 1992 to 11.0 g in 2002 in rural China [29,30]. Considering on the latency period between exposures and esophageal cancer onset, the increasing period effects from 2004 to 2008 might be related to the deteriorating dietary pattern of the Chinese rural population from 1992 to 2002, including decreased consumption of vegetables and fruits, and increased intake of pickled vegetables. Therefore, the trends of the period effects may be relevant to the changes in the dietary pattern of Chinese rural population.

In addition, it should be noted that after 2008, the ASMRs of esophageal cancer in rural China showed a downtrend again. There is no conclusive explanation for the cause of this change. However, the Four Trillion RMB (about 579 billion dollars) Investment Plan launched by the Chinese Central Government in 2008 has improved health service in rural China [32]. The Chinese government increased support for the construction of the three-level network of rural health service, especially provided more fund for the screening and early detection of esophageal cancer [33]. Therefore, we cautiously infer that the downtrends of the ASMRs may be associated with the Four Trillion RMB Investment Plan. Further studies are needed to understand other potential factors influencing this trend.

Our paper is the first study focusing exclusively on esophageal cancer mortality trends in rural China using APC analysis. The IE algorithm was applied to address the non-identification problem of the APC analysis. There are some specific limitations to our study. First, like other APC analyses of esophageal cancer [8,9,10,11,14], we analyzed the esophageal cancer mortality of total histologic types, including ESCC, EAC, and etc. However, the main risk factors for ESCC and EAC are different: the main risk factors for ESCC are smoking, alcohol consumption, and some particular dietary habits, while the main risk factors for EAC are adiposity, reflux esophagitis and Barrett’s esophagus. But, the esophageal cancer mortality of all histologic types in the WHO Mortality Database and the China Health Statistical Yearbook Database were only recorded as a whole, rather than being divided into different histologic types. However, ESCC is the predominant histologic type of esophageal cancer in rural China [2], which reduces the negative impact to some extent. Second, the CHIS reporting system is an annual disease surveillance system that was set up in 1973 and is based upon sample size of 10% the Chinese population [34]. Large swaths of rural China are not covered by cancer registries, which may impact the data quality. However, we believe that our data are reliable enough for the purposes of our study, which is the interpretation of general esophageal cancer mortality trends in rural China. Third, like other APC analyses, there was the inevitability of being affected by ecological fallacy since interpretations from results at population levels do not necessarily hold for individuals. Therefore, related hypotheses raised in this study still need further confirmation in the future individual-based studies.

## 5. Conclusions

In conclusion, we assumed that the general decrease in esophageal cancer mortality in rural China from 1989 to 2003 was caused by the downtrend of the cohort effects and period effects, while the general increase in mortality from 2004 to 2008 was caused by the uptrend of the period effects. The decrease in mortality after 2008 was related to the Four Trillion RMB Investment Plan launched by the Chinese Government. The declining cohort effects were probably related to the improvement of socioeconomic status in childhood and the decreasing consumption of alcohol and tobacco. The trends of the period effects were probably related to the changes in the dietary pattern of the Chinese rural population. The research results are beneficial for predicting future trend of esophageal cancer mortality in China, and providing some references to domestic and foreign esophageal cancer prevention.

## Appendix A

**Table A1:** Age-specific mortality rates for esophageal cancer by year of death in rural China (100,000).

Age	Year of Death									
	Rural Male					Rural Female				
	1989–1993	1994–1998	1999–2003	2004–2008	2009–2013	1989–1993	1994–1998	1999–2003	2004–2008	2009–2013
30–34	1.14	1.12	1.29	0.54	0.34	0.76	0.72	1.05	0.40	0.07
35–39	4.40	2.72	2.57	1.60	1.08	2.34	1.22	2.29	0.71	0.30
40–44	11.84	10.36	6.18	8.01	3.89	6.86	5.00	4.23	2.50	0.92
45–49	23.24	20.70	18.62	14.10	8.85	13.82	11.64	10.44	5.31	1.95
50–54	43.22	37.12	34.58	42.57	20.63	23.38	19.22	20.61	14.69	5.18
55–59	75.46	62.38	48.50	74.56	47.96	40.26	31.76	28.48	27.70	11.77
60–64	127.10	107.76	87.54	111.17	82.45	65.24	55.96	50.88	49.36	23.89
65–69	166.58	146.66	129.76	154.63	113.85	86.50	70.66	65.66	68.11	38.69
70–74	240.56	214.36	182.19	222.46	160.07	117.36	102.98	97.66	100.38	64.70
75–79	240.10	216.18	206.18	265.31	212.18	119.86	112.42	116.38	120.96	81.74
80–84	239.52	216.56	208.76	311.62	272.33	132.00	128.04	130.17	147.44	119.53

**Table A2:** Summary statistics comparing goodness-of-fit for different models.

Sex		Age Only	Age + Period	Age + Cohort	APC (IE)
Rural male	AIC	1187.63	625.10	565.88	6.58
	BIC	797.31	406.97	397.04	−99.26
Rural female	AIC	959.74	484.72	455.52	5.84
	BIC	587.98	289.65	319.61	−99.88

**Table A3:** APC model analysis results of esophageal cancer mortality in rural China.

	Rural Male		Rural Female	
	Coef.	SE	Coef.	SE
Age (year)				
30–34	−2.57	0.42	−2.22	0.50
35–39	−1.95	0.26	−1.83	0.35
40–44	−1.08	0.18	−1.14	0.25
45–49	−0.54	0.14	−0.60	0.19
50–54	0.01	0.11	−0.16	0.15
55–59	0.38	0.09	0.19	0.12
60–64	0.77	0.07	0.65	0.09
65–69	0.99	0.06	0.88	0.09
70–74	1.27	0.06	1.24	0.10
75–79	1.32	0.08	1.38	0.12
80–84	1.39	0.10	1.61	0.15
Period (year)				
1989–1993	0.01	0.06	0.19	0.09
1994–1998	−0.09	0.04	0.03	0.06
1999–2003	−0.15	0.03	0.01	0.04
2004–2008	0.19	0.04	0.06	0.06
2009–2013	0.05	0.06	−0.30	0.09
Cohort (year)				
1911–1915	0.70	0.15	0.53	0.27
1916–1920	0.73	0.13	0.65	0.25
1921–1925	0.76	0.12	0.73	0.25
1926–1930	0.77	0.11	0.79	0.24
1931–1935	0.71	0.11	0.81	0.25
1936–1940	0.58	0.12	0.75	0.26
1941–1945	0.42	0.14	0.68	0.28
1946–1950	0.31	0.16	0.56	0.30
1951–1955	0.27	0.18	0.43	0.33
1956–1960	0.12	0.20	0.17	0.36
1961–1965	−0.41	0.23	−0.36	0.41
1966–1970	−0.56	0.28	−0.58	0.48
1971–1975	−0.96	0.39	−0.92	0.64
1976–1980	−1.49	0.67	−1.48	1.04
1981–1985	−1.95	1.63	−2.77	3.63
